# Scd-1 deficiency promotes the differentiation of CD8^+^ T effector

**DOI:** 10.3389/fcimb.2024.1325390

**Published:** 2024-02-06

**Authors:** Yiwei Lin, Xushuo Li, Haojie Shan, Jie Gao, Yanying Yang, Linlan Jiang, Lu Sun, Yuwen Chen, Fangming Liu, Xiaowei Yu

**Affiliations:** ^1^ Department of Orthopaedic Surgery, Shanghai Sixth People’s Hospital Affiliated to Shanghai Jiao Tong University School of Medicine, Shanghai, China; ^2^ Jinshan Hospital Center for Tumor Diagnosis & Therapy, Jinshan Hospital, Fudan University, Shanghai, China; ^3^ Department of Oncology, Zhongshan Hospital, Fudan University, Shanghai, China; ^4^ Department of Physiology and Pathophysiology, School of Basic Medical Sciences, Shanghai Key Laboratory of Bioactive Small Molecules, Fudan University, Shanghai, China; ^5^ Department of Oncology, Shanghai Sixth People’s Hospital Affiliated to Shanghai Jiao Tong University School of Medicine, Shanghai, China; ^6^ Institute of Clinical Science, Zhongshan Hospital, Fudan University, Shanghai, China

**Keywords:** monounsaturated fatty acid, stearoyl-CoA desaturase, effector T cells, differentiation, infection

## Abstract

The impact of various fatty acid types on adaptive immunity remains uncertain, and their roles remain unelucidated. Stearoyl-CoA desaturase (Scd) is a Δ-9 desaturase, which is a key rate-limiting enzyme for the conversion of saturated fatty acids (SFA) to monounsaturated fatty acids (MUFA) in the fatty acid de novo synthesis. Scd-1 converts stearic acid (SA) and palmitic acid (PA) to oleic acid (OA) and palmitoleic acid (PO), respectively. In this study, through a series of experiments, we showed that Scd-1 and its resulting compound, OA, have a substantial impact on the transformation of CD8+ naïve T cells into effector T cells. Inactivation of Scd-1 triggers the specialization of CD8+ T cells into the Teff subset, enhancing the effector function and mitochondrial metabolism of Teff cells, and OA can partially counteract this. A deeper understanding of lipid metabolism in immune cells and its impact on cell function can lead to new therapeutic approaches for controlling the immune response and improving prognosis.

## 1 Introduction

Based on the length of the carbon chain and the number and position of unsaturated double bonds between two carbons, fatty acids are categorized as saturated fatty acids (SFA), monounsaturated fatty acids (MUFA), and polyunsaturated fatty acids (PUFA). Stearoyl-CoA desaturase (Scd) is a Δ-9 desaturase, which is a key rate-limiting enzyme for the conversion of SFA to MUFA in the fatty acid *de novo* synthesis (AM et al., 2017). Scd is divided into five isoforms based on the developmental stage and tissue distribution. Mice have four Scd isoforms (Scd-1,-2,-3, and -4), while humans have two isoforms (Scd-1 and -5), and Scd-1 is the predominant and highly expressed isoform (Castro et al., 2011; Hodson and Fielding, 2013). Scd-1 converts SFA into MUFA by transforming the carbon-carbon single bond between the ninth and tenth carbons at the carboxyl terminus into a carbon-carbon double bond. It converts stearic acid (SA) and palmitic acid (PA) to oleic acid (OA) and palmitoleic acid (PO), respectively.

Multiple studies have shown that fatty acids possess immunomodulatory properties and can modify the production and function of different elements of the immune system by altering the fluidity of membranes (Gutiérrez et al., 2019), lipid peroxidation (Zaloga, 2021), and the production of prostaglandins (Das, 2021). PA and linoleic acid stimulate cells to release inflammatory factors, whereas OA and docosahexaenoic acid (DHA) have anti-inflammatory effects (Rodriguez-Pacheco et al., 2017). Scd-1 is essential for maintaining fatty acid homeostasis as it converts SFA to MUFA via an oxygen-dependent reaction. Previous studies have linked the presence and function of Scd-1 to the emergence and progression of various ailments including diabetes, cancer, inflammatory bowel disease, obesity, fatty liver disease, and atherosclerosis. This establishes Scd-1 as a promising target for potential therapeutic interventions under these conditions (Zhang et al., 2014; AM et al., 2017). Humoral immunity is strongly linked to MUFA, and the product of Scd-1, OA, is crucial for the proliferation of B cells, antibody class switching, and maintenance of appropriate mitochondrial metabolism (Zhou et al., 2021). Moreover, Scd-1 has a substantial impact on T cell fate and autoimmunity. According to recent research, Scd1 deficiency promotes the hydrolysis of triglycerides and phosphatidylcholine; this increases the release of DHA to activate the nuclear receptor peroxisome proliferator-activated receptor gamma (PPARγ), which promotes regulatory T cell differentiation and thereby reduces the severity of multiple sclerosis (Grajchen et al., 2023). It has also been shown that inhibition of Scd-1 stimulates CD8^+^ T cells and enhances IFN-γ production and cytotoxic activity of T cells. In mouse tumor models, Scd-1 inhibitors synergistically enhanced the anti-tumor effects of anti-PD-1 antibody therapy and CAR-T cell therapy(Katoh et al., 2022; Sugi et al., 2023). Dietary intervention or drugs targeting Scd-1 to regulate MUFA may be beneficial in the treatment of certain tumors, infectious diseases, and autoimmune disorders.

Sepsis is considered a biphasic disease in which the immune-activated and immune-suppressed phases can occur simultaneously (Nedeva et al., 2019), and despite the fact that sepsis has been well understood, it remains a worldwide health challenge. During the initial phase of hyperinflammation, known as the “cytokine storm”, the innate immune system releases a large number of inflammatory molecules (Delano and Ward, 2016). Subsequently, lymphoid and myeloid lineage cells begin to fail and the body assumes an immunocompromised, low-inflammatory state (Boomer et al., 2014). During the initial hyperinflammatory response phase of the disease, the acute innate immune response is triggered immediately, while the adaptive immune system begins to fine-tune the antigen-specific response. Nowadays, there are more and more studies focusing on the T-cell response in the acute adaptive immune response in sepsis (even within a few hours). The endotoxemia model, as a disease model of systemic acute inflammatory response, correlates well with sepsis, albeit with some limitations(Lewis et al., 2016). In this study, we focused on the role of Scd-1-deficient CD8^+^ T cells in the endotoxemia and LCMV-infection, hoping to provide new ideas for therapies to improve immune system homeostasis.

Here, we investigated the effects of Scd-1 and its derivative OA on the differentiation, functionality, and metabolic state of CD8^+^ T cells, which play pivotal roles in adaptive immunity. Our evidence suggests that there are variations in the lipid composition among CD8^+^ naïve T cells (Tnaïve), effector T cells (Teff), and memory T cells (Tmem). Specifically, Teff cells exhibit elevated levels of OA, PO, and Scd-1, which account for their production. Scd-1 knockout promotes CD8^+^ T cell differentiation into the Teff subset, enhancing effector function and mitochondrial metabolism. To a certain extent, OA can reverse the phenomena described above. In the LCMV infection model, Scd-1 deficiency enhances resistance to viral infection. However, in animal models of endotoxemia, OA has been shown to partially hinder the development of CD8^+^ T cells into the Teff subpopulation, decrease the expression of Teff effector molecules, diminish inflammation, and alleviate the symptoms of endotoxemia.

## 2 Materials and methods

### 2.1 Ethics statement

The animal study was reviewed and approved by Zhongshan Hospital, Fudan University Ethical Committee (No. 2023-040).

### 2.2 Animals

B6.129-Scd1^tm1Ntam^/J mice (*Scd-1* ^-/-^ mice) were donated by Dr. Zhinan Yin from the Biomedical Translational Research Institute, Faculty of Medical Science, Jinan University, China. C57BL/6J mice were purchased from JiesI Jie Laboratory Animal Co., Ltd., China. All mice were kept in specific pathogen-free rooms at a constant temperature of 23°C with a 12-hour light cycle. Male mice aged 6–8 weeks were used for the experiments.

### 2.3 Cell culture

The cell culture method is described in detail in Lu et al. (2021). The spleen from the mouse was extracted, pulverized in PBS, and subsequently passed through a 70 μm filter. After being spun at 1500 rpm for 5 min, the cells were suspended in 2–3 mL buffer to lyse the red blood cells, left to incubate at 0°C for 6 min, and then washed twice. A MojoSort Mouse CD8 T Cell Isolation Kit (BioLegend) was used to screen the acquired CD8^+^ T cells. The cells were resuspended in complete medium (RPMI medium 1640 with 10% FBS, 1% penicillin/streptomycin, 2.5% HEPES, 1% sodium pyruvate, 1% nonessential amino acids, and 0.1% 2-ME) at a concentration of 1.5 × 10^6^ cells/mL and placed in a 24-well plate at 1 mL per well. The cells were stimulated with 5 μg/mL of anti-CD3 and 0.5 ug/mL of anti-CD28 in a 37°, 5% CO_2_ incubator for three days. In addition, 100 U/mL IL-2 was also added to the samples. Teff cells were obtained by stimulating the cells with CD3/28 for three days followed by washing with PBS to eliminate the CD3/28. Then, IL-2 was induced for an additional three days. Cell counting was performed daily and the complete medium was replaced on a daily basis. The fatty acid concentrations and times used for cell culturing were obtained from Zhou et al. (2021). Cultured CD8^+^ T cells were stimulated with CD3/28 for 3 days and subsequently induced with IL-2. Then, the corresponding fatty acids were added and the sample cultured for another 72 h. There were four groups that required additional fatty acids and the following concentrations were added: OA, 100 μM; SA, 25 μM; PO, 25 μM; and PA, 100 μM.

### 2.4 Fatty acid measurement of CD8^+^ T cells

The cells were collected and rinsed three times with PBS. Then, the specimens were frozen in liquid nitrogen followed by thawing at 37°C, which was repeated for 4–5 cycles. Pre-cooled isopropanol (HPLC grade) and the mass-spec standard were added to each sample tube. The mixture was thoroughly mixed, incubated for 10 min at room temperature, and refrigerated at –20°C overnight. The samples were then spun at 12,000 rpm for 20 min and 200 μL of the supernatant was removed for further use. A total of 10 μL from each sample were mixed to create a quality control sample. The data was obtained after performing five consecutive stable injections of the quality control samples. After completing a set of ten samples, quality control sample detection was performed to assess the collection parameters. A lipidomics analysis of the CD8^+^ T cells was performed using a QTRAP 5500 (AB SCIEX) LC-MS/MS system and a Waters Acquity UPLC BEH HILIC column was used for chromatographic separation. An internal standards kit (AB SCIEX) was added to each sample. There was 95% acetonitrile containing 10 mmol/L ammonium acetate in phase A and 50% acetonitrile containing 10 mmol/L ammonium acetate in phase B. The mobile phase gradient elution was as follows: the B phase began at 0.1% and increased to 20% for 10 min, then to 98% over 1 min, which was held for 2 min, with a final decrease to 0.1% over 1 min, which was held for 2 min. The parameters were as follows: the curtain gas, GS1, and GS2 were set to 35, 50, and 60 psi, respectively; the ion spray voltage was set to 5,500 V; and the declustering, entrance, and collision voltages were maintained at 80, 10, and 50 V respectively. SCIEX Analyst software (version 1.7) was used to collect the data and MultiQuant software (AB SCIEX) was used to process the data after mass spectrometry identification of lipids,.

### 2.5 Flow cytometry

The cells were stained with fixable viability stain 510 (BD Horizon) in PBS at a ratio of 1:500 at 4°C for 30 min. Following two washes with PBS, the cells were subjected to staining with antibodies targeting surface markers at a dilution of 1:200, maintained at 4° for 30 min. For intracellular staining, cells were incubated with antibodies for 30 min at 4°C following permeabilization using FACS permeabilization buffer (Fixation/Permeabilization, BD Biosciences). Stimulation with cell activation cocktail (with Brefeldin A, Biolegend) for 4-6 hours before measurement of T cell effector molecules (GZMA, GZMB, GZMC, PRF, IFN-γ). **Supplementary Table 1** contains information about the fluorochromes and clones. The cells were stained with TMRM and BODIPY (Invitrogen) at ratios of 1:1000, for 30 min at room temperature. Finally, the samples were analyzed using a BD FACS Aria III flow cytometer and the resulting data were analyzed using FlowJo10 software.

### 2.6 Quantitative real-time PCR

Total mRNA was obtained from CD8^+^ T cells using TRIzol reagent. Subsequently, Superscript II (Invitrogen) was used to produce cDNAs via reverse transcription according to the manufacturer’s instructions. The SYBR Green-based method was used for three rounds of PCR on each sample. The procedure involved warming at 95°C for 15 s and then lowering the temperature to 60°C for 1 min. This process was repeated 40 times. The mRNA relative expression levels were determined using the 2^-ΔΔCT^ method and standardized to GAPDH. The primers used in this study are listed in **Supplementary Table 2**.

### 2.7 RNA-seq and analysis

CD8^+^ Teff cells were used to extract total RNA using an RNeasy mini kit (Qiagen). The total RNA was then converted into strand-specific libraries using a TruSeq Stranded Total RNA Sample Preparation kit (Illumina) according to the manufacturer’s instructions. The libraries were measured using a Qubit 2.0 Fluorometer (Life Technologies) and confirmed using an Agilent 2100 bioanalyzer (Agilent Technologies). Clusters were produced using cBot and subsequently sequenced using an Illumina NovaSeq 6000 (Illumina). HISAT2 (v2.0.477) was used to map RNA-seq reads from each sample to the reference genome (ftp://ensemblgenomes.org/). StringTie (v.1.3.0) was used to sequence the read calculations, followed by normalization using the Trimmed Mean of M values (TMM) method and conversion to FPKM. The edgeR package in R was used to analyze the variance in gene expressions between groups and differentially expressed genes (| log2 fold change | ≥ 1.0, *p* < 0.001) were subsequently examined. 2.8 Metabolism assay

Cultured CD8^+^ Teff cells were assessed for OCR using a Seahorse XF mitochondrial stress test kit (Agilent) on an XFe96 Extracellular Flux Analyzer (Agilent). Seahorse XF DMEM (Agilent) was used to replace the previously prepared culture medium. It was then supplemented with pyruvate, glutamine, and glucose at 1% concentrations. At least four samples per group were seeded onto a 96-well plate coated with poly-D-lysine at a density of 1.5×10^5^ cells per well. The CD8^+^ Teff cells mitochondrial respiration was measured using the Seahorse XFe96 after being incubated at 37° in a CO_2_-free incubator for 60 min according to the manufacturer’s instructions. Basal metabolism, ATP production, maximum respiration, and spare respiratory capacity were determined based on the OCR graphs (Brand and Nicholls, 2011; Chowdhury et al., 2018).

### 2.9 Endotoxemia animal model

Four groups were formed: the WT endotoxemia group, WT endotoxemia group receiving OA treatment, SCD-1^-/-^ endotoxemia group, and SCD-1^-/-^ endotoxemia group receiving OA treatment. Each group contained five mice. All mice were intraperitoneally injected with 3 mg/kg lipopolysaccharide (LPS, O55:B5, Sigma-Aldrich) and harvested 48 h later. Throughout the experiment, the weight of the mice was recorded every 12 h and their condition was assessed using the murine sepsis score (MSS) method outlined by Shrum et al. (2014). Referring to the work of Terés et al.(Terés et al., 2008) and Medeiros-de-Moraes et al.(Medeiros-de-Moraes et al., 2018), the mice were administered oral gavage for 14 days prior to establishment of the LPS-induced endotoxemia model. Each mouse in the OA treatment group received a gavage of a solution containing 0.28 mg OA in 100 μL on a daily basis, whereas mice in the remaining groups were administered 100 μL of physiological saline by gavage on a daily basis.

### 2.10 LCMV-infected animal model

Four groups were formed: PBS injection WT group, LCMV injection WT group, PBS injection *Scd-1* ^-/-^ group, and LCMV injection *Scd-1* ^-/-^ group. Each group contained five mice. Briefly, 8-week-old mice were intraperitoneally injected with 2×10^5^ plaque-forming units of LCMV Armstrong. On day 7 after the injection, the mice were sacrificed for the following flowcytometry (FCM) analysis and the spleen virus copies number were detected by RT-qPCR using specific primers probing the LCMV-Armstrong viral RNA. Total RNA was isolated using RNA-Quick Purification Kit (ES Science, RN001). RNA (500 ng) was reverse transcribed into cDNA using 5× PrimeScript RT Master Mix(Takara, Cat# RR036A).The relative expression of gene transcripts compared to the control 18S rRNA (Beyotime, QM00046M).

### 2.11 ELISA

After the animals have been harvested, peripheral blood was obtained using the orbital blood collection method. The collected blood was centrifuged at 3,000 rpm for 10 min and the supernatant was collected. Commercial enzyme-linked immunosorbent assay kits (R&D Biosystems) were used to measure the TGF-α, IL-1β, IL-6, and IL-10 levels in the serum according to the manufacturer’s instructions.

### 2.12 Statistical analysis

Data were statistically analyzed using GraphPad Prism (version 8). The mean ± SEM of at least three independent experiments are given for each set of experimental results. Student’s t-test was used to compare two groups and one-way analysis of variance (ANOVA) was used to compare several groups. Statistically significant results were those with P-values less than 0.05. Values of P < 0.05 were ranked as *P < 0.05, **P < 0.01, and ***P < 0.001.

## 3 Results

### 3.1 Different CD8^+^ T cell subsets differ in lipid composition and scd-1 expression

**Figure 1:**
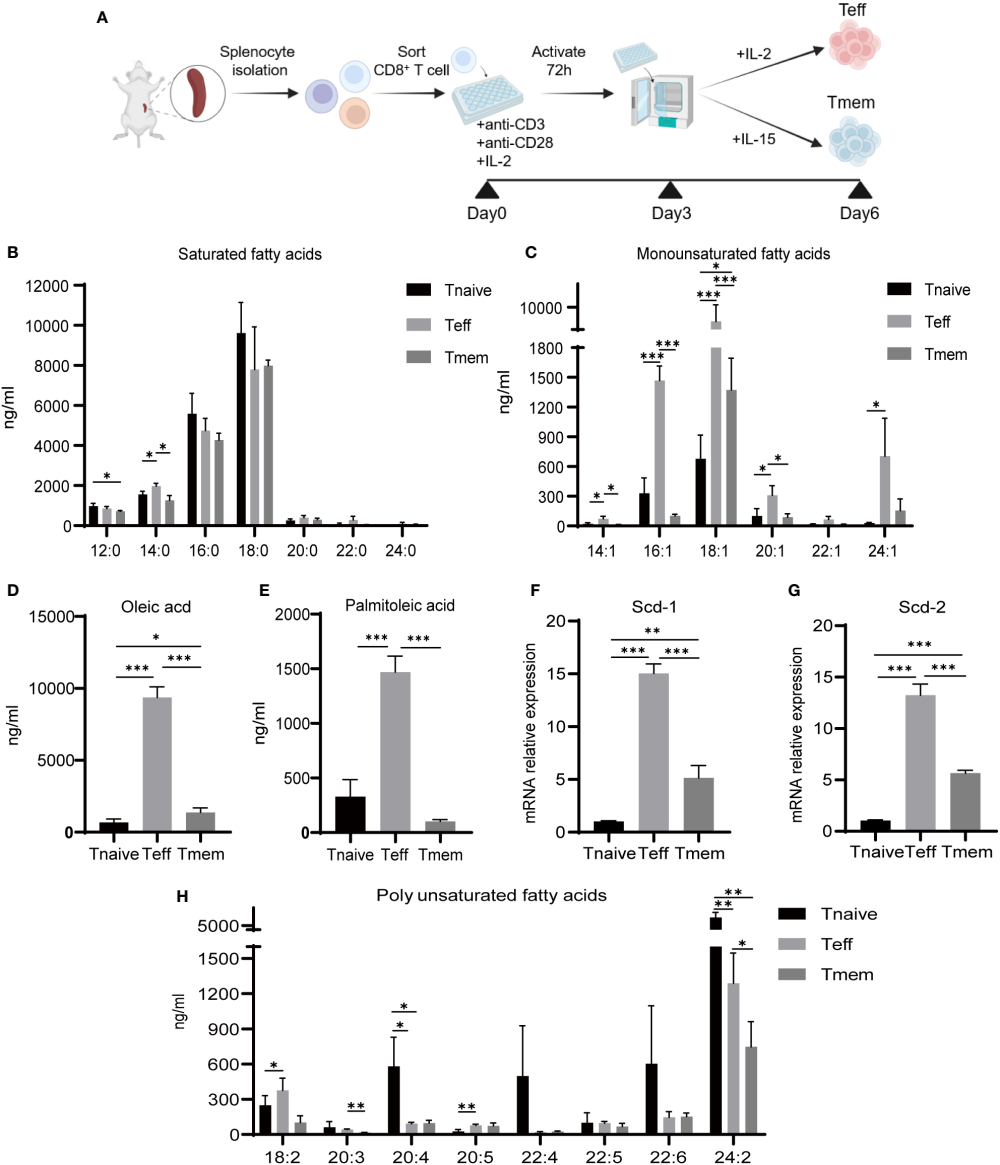
Content of fatty acids and corresponding enzyme expression in CD8^+^ Tnaive, Teff and Tmem. **(A)** Illustration of the culture method for CD8^+^ Teff and Tmem cells *in vitro*. **(B, C)** Distribution of saturated and monounsaturated fatty acids in CD8^+^ T cell subsets (n=3). **(D, E)** Distribution of OA and PO in CD8^+^ T cell subsets (n=3). **(F, G)** mRNA expression of Scd-1 and Scd-2 in CD8^+^ T cell subsets (n=3). **(H)** Distribution of polyunsaturated fatty acids in CD8^+^ Tnaive (CD44^lo^ CD62L^hi^), Teff (CD44^hi^ CD62L^lo^), and Tmem (CD44^hi^ CD62^hi^) cell subsets (n=3). The results presented as mean + SEM. *, P<0.05; **, P<0.01; ***, P<0.001.

Initially, we used magnetic beads to sort CD8^+^ T cell from spleens of WT mice, then obtained Tnaive, Teff, and Tmem, as described in methods (**Figure 1A**, **Supplementary Figure 1**). Liquid chromatography-mass spectrometry (LC-MS) was used to ascertain the lipid makeup of CD8^+^ Tnaïve, Teff, and Tmem. No notable disparities in SFA content among the three cellular groups were observed; however, exceptionally minor variations in the quantities of lauric acid (C12:0) and myristic acid (C14:0) were observed (**Figure 1B**). Nevertheless, the MUFA content exhibited notable variation among the three cell groups (**Figure 1C**). In our study, we found that Teff cells had a higher amount of MUFA than Tnaïve and Tmem cells, particularly in terms of OA (C18:1) and PO (C16:1) (**Figures 1C–E**). In addition, Teff had higher levels of myristelaidic acid (C14:1), 11(Z)-eicosenoic acid (C20:1), and nervonic acid (C24:1) than Tnaïve and Tmem (**Figure 1C**). The expression of scd-1 and -2 mRNA, which are responsible for the production of OA and PO, respectively, was identified in various T cell subsets. Compared with Tnaïve and Tmem, Teff exhibited elevated levels of scd-1 and -2, elucidating the reason behind the disparity in OA and PO quantities among these subtypes (**Figures 1F, G**). The three subtypes also exhibited notable disparities. In general, Tnaïve had a higher PUFA content than Teff and Tmem (**Figure 1H**). Considering that OA and PA are the prevailing MUFA in cells (Jump, 2004), we hypothesized that their notable disparity might result in alterations in cellular function and metabolism.

### 3.2 Knocking out scd-1 affects the differentiation and functionality of CD8^+^ T cells *in vivo*

**Figure 2:**
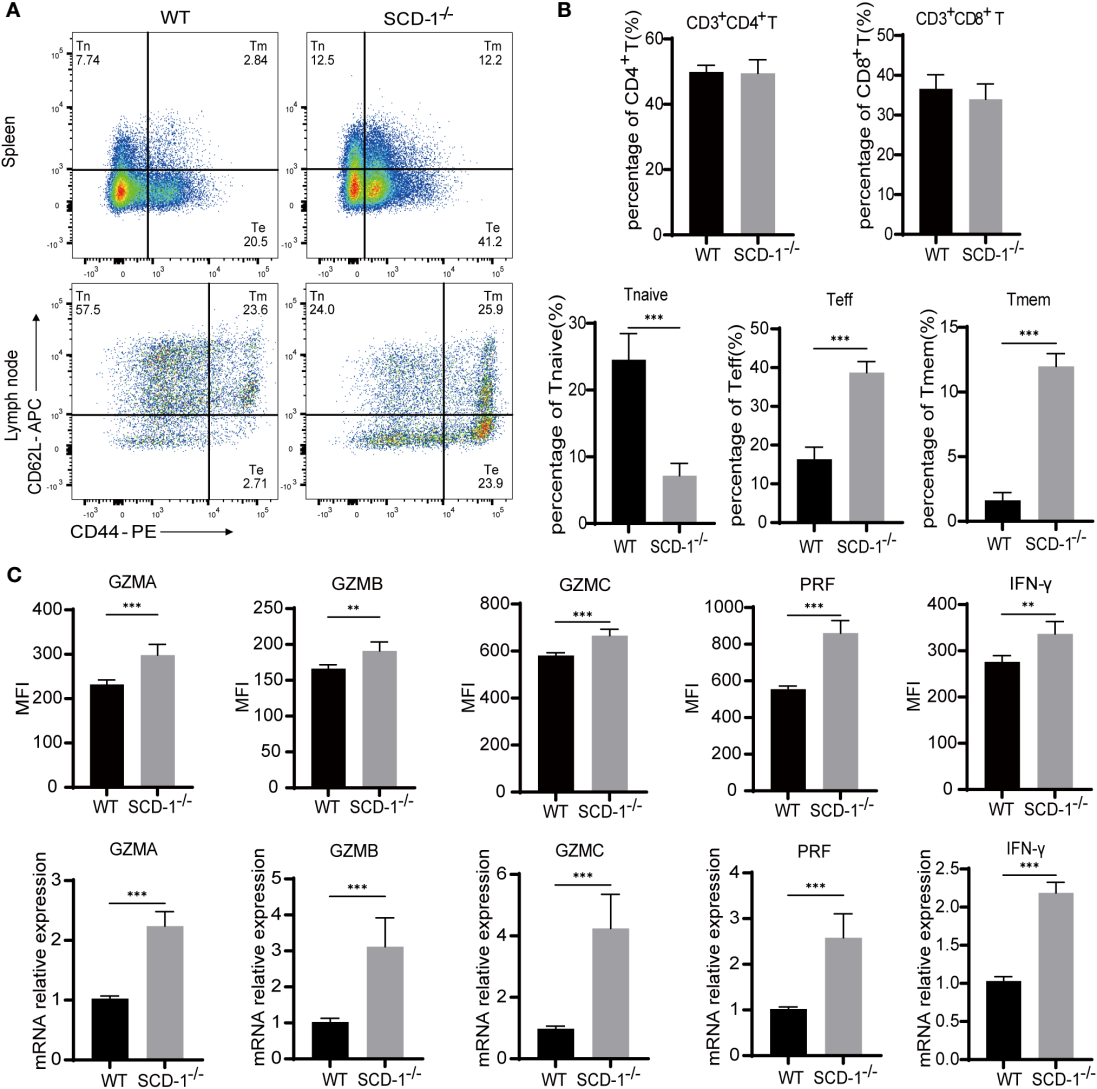
Knocking out Scd-1 affects the differentiation and function of CD8^+^ T cells. **(A)** Flow cytometry analysis of CD8^+^ T cell subsets in the spleen and lymph nodes of WT and *Scd-1^-/-^* mice. **(B)** Frequencies of CD3^+^ CD4^+^ T cells, CD3^+^ CD8^+^ T cells, Tnaive, Teff, and Tmem cells in the spleen and lymph nodes of WT and *Scd-1^-/-^* mice (n=5). **(C)** Statistical graphs of flow cytometry and rt-PCR of GZMA, GZMB, GZMC, PRF, IFN-γ expression (n=5). The results presented as mean + SEM. **, P<0.01; ***, P<0.001.

To further examine the effect of Scd-1 on the differentiation of CD8^+^ T cells, we compared CD8^+^ T cell subsets in the spleen and lymph nodes of mice with and without Scd-1 (Knockout of Scd-1 in CD8^+^ T cells has been validated, **Supplementary Figure 2A**). CD3^+^ CD8^+^ T cells were classified into the following distinct subsets: Tnaïve (CD44^lo^ CD62L^hi^), Teff (CD44^hi^ CD62L^lo^), and Tmem (CD44^hi^ CD62^hi^). The gating steps of flow cytometry have been shown in **Supplementary Figure 2B**. Consistent findings were observed in the spleen and lymph nodes of both WT and *Scd-1* ^-/-^ mice. The ratios of CD3^+^ CD8^+^ and CD3^+^ CD4^+^ T cells in lymphocytes were nearly identical in both mouse genotypes. The percentage of CD3^+^ CD8^+^ Tnaïve cells was much higher in the WT group than in the *Scd-1* ^-/-^ group, whereas the *Scd-1* ^-/-^ group had a higher percentage of Teff and Tmem cells than the WT group (**Figures 2A, B**). Dendritic cells, neutrophils, macrophages, monocytes, and NK cells did not differ in proportion in the spleens of WT and *Scd-1* ^-/-^ mice (**Supplementary Figure 2D**). T cells were stimulated with cell activation cocktail (with Brefeldin A) for 4-6 hours to measure the expression and secretion of effector molecules. The *Scd-1* ^-/-^ group exhibited higher expression of effector molecules like granzyme A (GZMA), granzyme B (GZMB), granzyme C (GZMC), perforin (PRF), interferon-γ (IFN-γ) compared with the WT group at both mRNA and protein levels (**Figure 2C**, **Supplementary Figure 2C**). Consistent with this, the work of Sugi et al. (2023) also demonstrated that inhibition of Scd-1 could potentiate IFN-γ production and the cytotoxic activity of CD8^+^ T cells. These results indicated that the loss of Scd-1 in CD8^+^ T cells causes a tendency to differentiate into the Teff subtype.

### 3.3 Knocking out scd-1 affects the expression of genes related to CD8^+^ Teff differentiation and function *in vitro*

**Figure 3:**
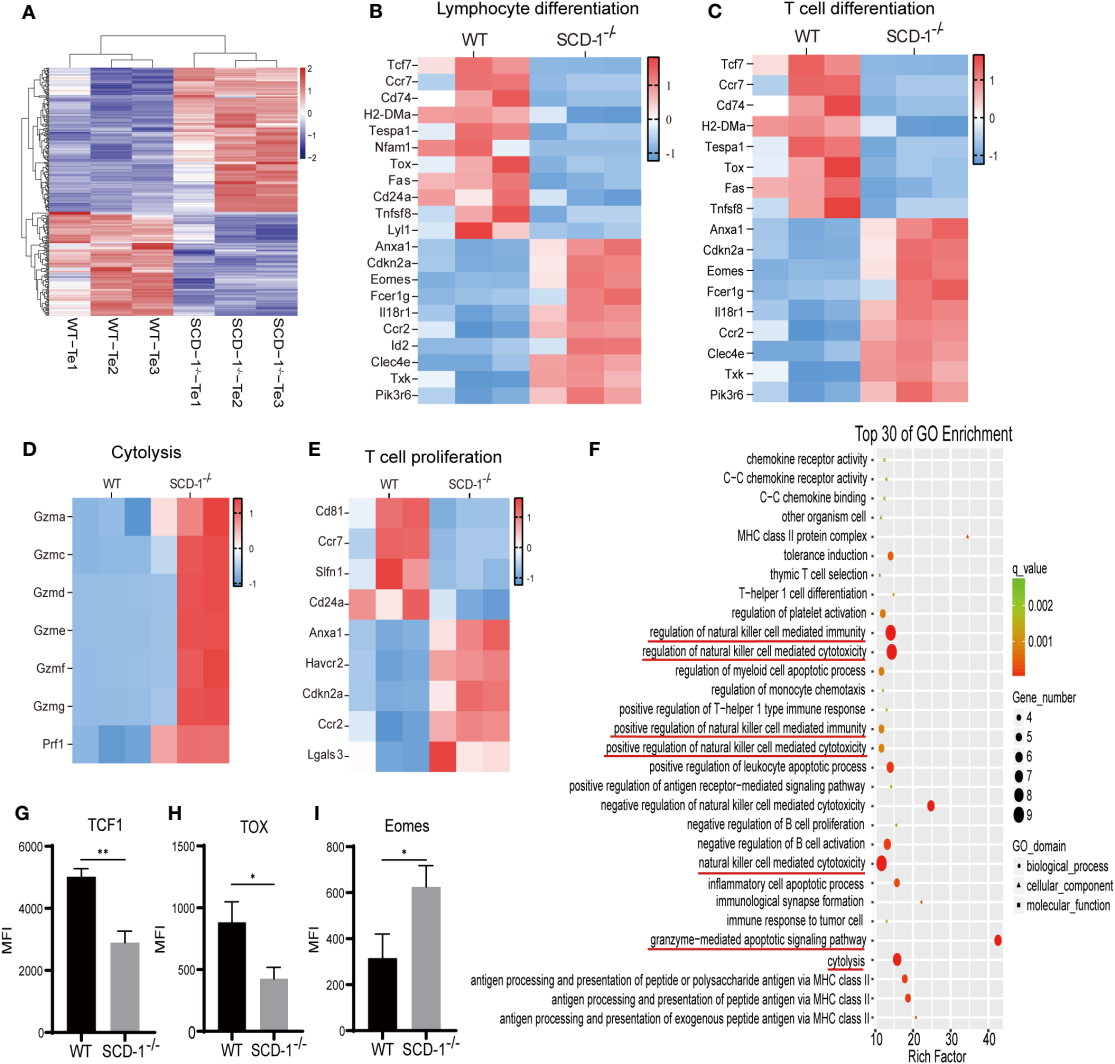
Differential gene expression of Teff between WT and *Scd-1* ^-/-^ mice. **(A)** Heatmap of differentially expressed genes in Teff between WT and *Scd-1* ^-/-^ mice (n=3). **(B-E)** Differential gene expression heatmaps of Teff in lymphocyte differentiation-related genes, T cell differentiation, cytolysis, and T cell proliferation-related genes between WT and *Scd-1* ^-/-^ mice, respectively (n=3). **(F)** GO enrichment analysis of differentially expressed genes in Teff between WT and *Scd-1* ^-/-^ mice (n=3). **(G-I)** Flow cytometry analysis of Tcf1, Tox, and Eomes in Teff between WT and *Scd-1* ^-/-^ mice, respectivel (n=3). The results presented as mean + SEM. *, P<0.05; **, P<0.01.

To further explore the influence of Scd-1 on the differentiation and functional status of Teffs, we isolated CD8^+^ T cells from the spleens of both WT and *Scd-1* ^-/-^ mice using a kit, which were then induced to differentiate into Teff cells using IL-2 after three days of stimulation with CD3/28 (**Figure 1A**). Next, we performed RNA transcriptome sequencing (**Figure 3A**). We found that differentially expressed genes in Teffs between WT and *Scd-1^-/-^* were enriched in cell differentiation, cytolysis, and proliferation (**Figures 3B–E**). In terms of cell differentiation, WT Teff expressed higher levels of *Tcf7*, *Ccr7*, *Cd74*, *Tox*, *Tespa1*, and *Fas* than *Scd-1^-/-^* Teff, while decreasing the expression of *Anxa1*, *Eomes*, *Clec4e*, and *Pik3r6* (**Figures 3B, C**). In terms of killing target cells with effector molecules (i.e., cytotoxicity of effector T cells), Teff in the *Scd-1^-/-^* group expressed more granzymes and perforin (*Gzma*, *Gzmc*, *Gzmd*, *Gzme*, *Gzmf*, *Gzmg*, *and Prf1*) than Teff in the WT group (**Figure 3D**). The expression of *Anxa1*, *Havcr2*, *Cdkn2a*, *Ccr2*, and *Lgals3* in the Teffs of *the Scd-1* ^-/-^ group, genes associated with cell proliferation, was higher than that in the WT group. Conversely, *Cd81*, *Ccr7*, *Slfn1*, and *Cd24a* expression was decreased (**Figure 3E**). In the GO enrichment analysis of differentially expressed genes, the differentially expressed gene sets between the WT and *Scd-1* ^-/-^ groups were mainly related to the regulation of natural killer cell-mediated immune activity and its related cytotoxic effects, as well as to granzyme-mediated cell apoptosis and cytolysis (**Figure 3F**). This also demonstrates that the knockout of Scd-1 enhances the effector function of Teff. The expression of Tcf1 (encoded by *Tcf7*), Tox, and Eomes in Teff cells of the WT and *Scd-1* ^-/-^ groups was detected by flow cytometry and was consistent with the RNA-seq results. Compared with Teff cells in the WT group, Teff cells in the *Scd-1* ^-/-^ group exhibited reduced Tcf1 expression and elevated Eomes and Tox expression (**Figures 3G–I**).

### 3.4 OA addition partially reverse effector differentiation and function in *Scd-1* ^-/-^ Teff

**Figure 4:**
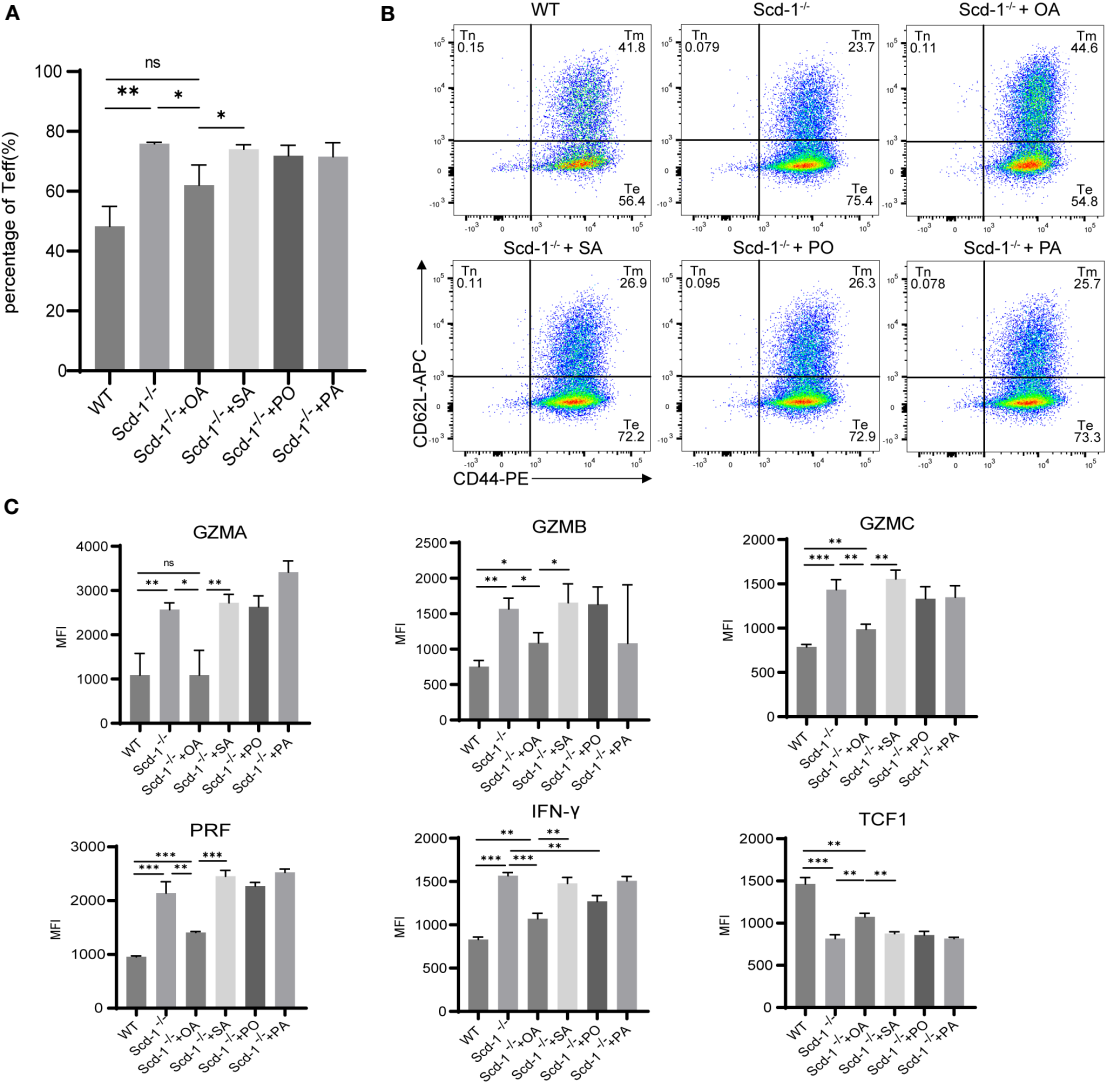
Changes in the subset proportions and functional status of Teff in the *Scd-1* ^-/-^ group after supplementation with OA, SA, PO, and PA. **(A, B)** Flow cytometry and statistical analysis of Teff subsets in WT, *Scd-1* ^-/-^ groups before and after supplementation with OA, SA, PO, and PA (n=3). **(C)** Flow cytometry graphs showing the expression changes of GZMA, GZMB, GZMC, PRF, IFN-γ, and Tcf1 in WT Teff, *Scd-1* ^-/-^ Teff before and after replenishment with OA, SA, PO, and PA (n=3). The results presented as mean + SEM. *, P<0.05; **, P<0.01; ***, P<0.001. ns, not significant.

Since Scd-1 is the key limiting enzyme that catalyzes the conversion of SA and PA into OA and PO, respectively, it was confirmed that knocking out Scd-1 would make CD8^+^ T cells more inclined to differentiate toward the Teff subtype in the previous section. To investigate whether changes in the differentiation and functional state of CD8^+^ T cells caused by Scd-1 deficiency were reversible, we introduced four fatty acids (OA, SA, PO, and PA) for incubating *Scd-1* ^-/-^ Teff cells. Throughout this process, we closely monitored their differentiation and functional states. CD8^+^ T cells were isolated using a kit and stimulated with CD3/28 and IL-2 for three days, then induced with IL-2 for another three days, we found that the proportion of the Teff subgroup (CD44^hi^ CD62^lo^) in the *Scd-1* ^-/-^ group was greater than that in the WT group. Additionally, introducing OA into the *Scd-1* ^-/-^ group resulted in a reduction in the proportion of the Teff subgroup (**Figures 4A, B**). However, the addition of different fatty acids did not alter the Teff clustering ratio, including PO, which isalsoa product of Scd-1 (**Figures 4A, B**).

The relationship between Tcf1 and T-cell differentiation is very close, with high expression in Tnaive and Tmem cells and lower expression in Teff cells (Raghu et al., 2019; Zhang et al., 2021). Adding OA to Teff from the *Scd-1* ^-/-^ group resulted in a reduction in the levels of effector molecules GZMA, GZMB, GZMC, PRF, and IFN-γ, while simultaneously enhancing the expression of Tcf1 (**Figure 4C**). However, supplementing SA, PO, and PA to Teff from the *Scd-1* ^-/-^ group did not decrease the expression of effector molecules, except for PO which reduced IFN-γ expression (**Figure 4C**). Furthermore, only OA could slightly increase the expression of Tcf1 in Scd-1^-/-^ Teff cells. In summary, OA can partially reverse alterations in the differentiation and functional condition of CD8^+^ T cells that arise from the absence of Scd-1, whereas SA, PO, and PA lack the ability to reverse this situation.

### 3.5 Scd-1 deletion affects the metabolism of CD8^+^ Teff and can be reversed with OA

**Figure 5:**
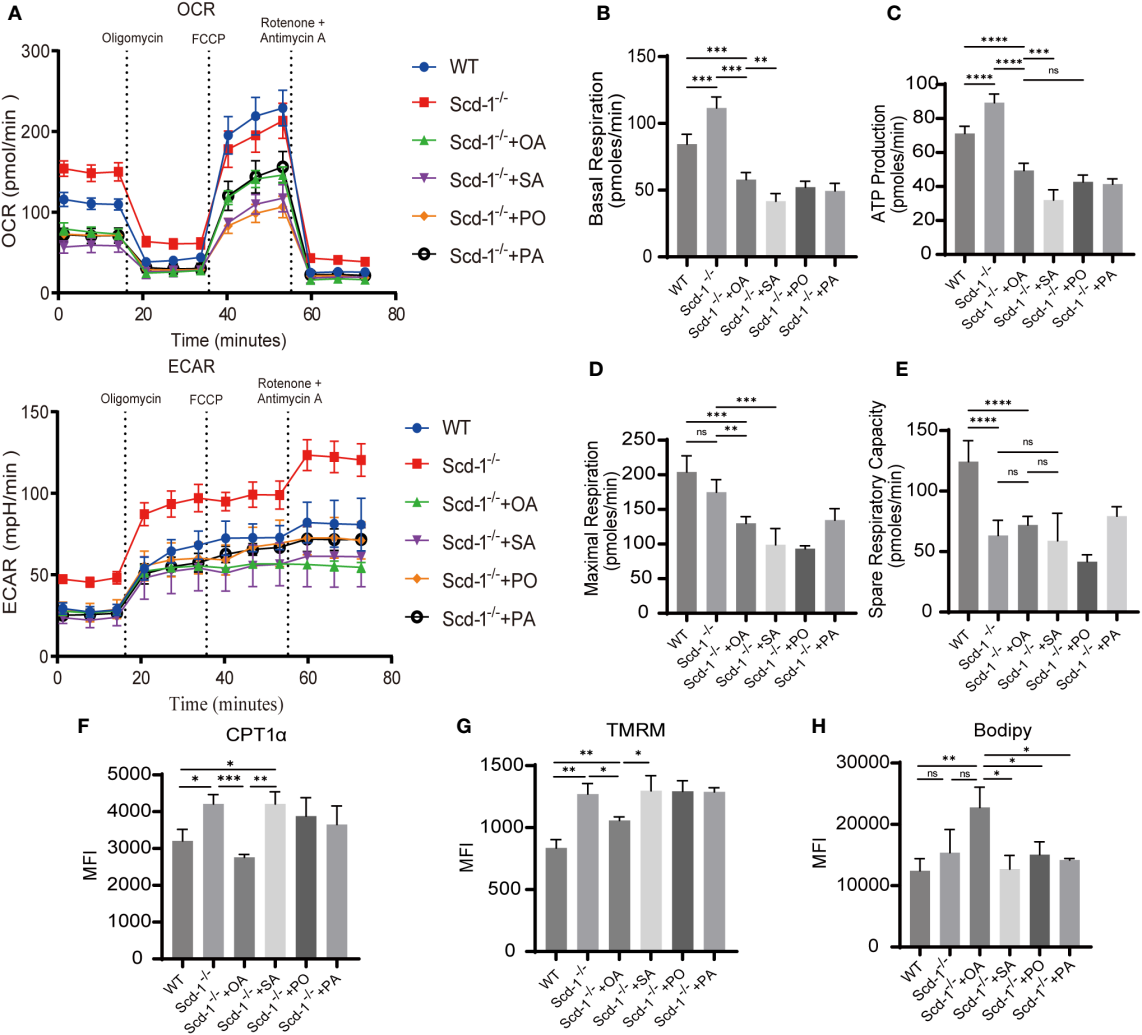
Knocking out Scd-1 affects the metabolism of CD8^+^ Teff and can be reversed with OA. **(A)** Mitochondrial stress experiment in Teff cells of WT, Scd-1^-/-^, and Scd-1^-/-^ supplemented with OA, SA, PO, and PA (n=3~7). **(B-E)** The statistical graphs of basal respiration, ATP production, maximum respiration, and spare respiratory capacity for the Teff in the WT group, Scd-1^-/-^ group, and after replenishment with OA, SA, PO, and PA (n=3~7). **(F-H)** Flow cytometry data analysis of CPT1α expression, mitochondrial membrane potential, and lipid content in Teff from WT, Scd-1^-/-^, and after replenishment with OA, SA, PO, and PA (n=3). The results presented as mean + SEM. *, P<0.05; **, P<0.01; ***, P<0.001. ns, not significant.

Because cellular energy utilization and metabolism are closely related to their differentiation and function, we measured the oxygen consumption rate (OCR) and extracellular acidification rate (ECAR) of Teff cells in the WT and *Scd-1* ^-/-^ groups. This assay was performed before and after replenishing the cells with OA, SA, PO, or PA. When Tnaïve is transformed into Teff, the levels of oxidative phosphorylation and glycolysis increase (Sukumar et al., 2013). The findings revealed that the *Scd-1* ^-/-^ group exhibited elevated resting basal respiration compared to the WT group, whereas the Teff of *Scd-1* ^-/-^ demonstrated a decline in basal respiration following the replenishment of OA, SA, PO, and PA (**Figures 5A, B**). This suggests that the mitochondrial metabolism of *Scd-1* ^-/-^ Teffs was enhanced and the addition of fatty acids inhibited this elevated mitochondrial state. *Scd-1* ^-/-^ mice exhibited greater ATP synthesis than WT mice. Following the addition of OA, SA, PO, or PA, ATP synthesis in *Scd-1* ^-/-^ Teff cells decreased (**Figures 5A, C**). This suggests that Scd-1^-/-^ Teff may require more ATP production to generate more effector molecules (GZMA, GZMB, GZMC, PRF, IFN-γ) than WT Teff. Maximal respiration did not show any notable variation between Teff cells from *Scd-1* ^-/-^ and WT groups, suggesting that both groups possess a comparable ability to utilize mitochondrial respiration under stressful conditions. However, the inclusion of fatty acids while cultivating Teffs from the *Scd-1* ^-/-^ group reduced the maximal respiration rate (**Figures 5A, D**). Teff of the *Scd-1* ^-/-^ group had weaker spare respiratory capacity (SRC) in comparison to that of the WT group. Despite the inclusion of OA, SA, PO, and PA in the Teff of the *Scd-1* ^-/-^ group, SRC remained unchanged (**Figures 5A, E**). This suggests that Teff in the WT group has better metabolic adaptability and stress resistance than Teff in the *Scd-1* ^-/-^ group. We also observed that knockdown of Scd-1 resulted in enhanced glycolysis in CD8^+^ T cells, whereas supplementation with OA decreased glycolysis (**Figure 5A**). Since the induction of high glycolytic activity in CD8^+^ T cells facilitates the differentiation of CD8^+^ T cells to Teff, and the down-regulation of glycolytic levels is detrimental to the production of relevant effector molecules such as IFN-γ in Teff (Cao et al., 2023). This is supported by the fact that deletion of Scd-1 in CD8^+^ T cells leads to a tendency to differentiate into the Teff subtype.

Carnitine palmitoyltransferase 1 alpha (CPT1α) plays a crucial role as a restricting enzyme in the fatty acid oxidation pathway. CPT1α primary function involves the transport of fatty acids to the mitochondria, where they undergo breakdown and metabolism, ultimately boosting mitochondrial oxidative phosphorylation (Bonnefont et al., 2004). After the inclusion of OA, the *Scd-1* ^-/-^ group exhibited a decrease in the expression of CPT1α, which was initially higher than that of the WT group (**Figure 5F**). This aligns with the previously noted occurrence of increased basal respiration and ATP generation in the Teffs of the *Scd-1* ^-/-^ group, as opposed to the WT group. Notably, Teff in the *Scd-1* ^-/-^ group had a higher mitochondrial membrane potential than that in the WT group (**Figure 5G**). The presence of OA resulted in a reduction in mitochondrial membrane potential, whereas the presence of SA, PO, and PA did not have any effect (**Figure 5G**). This suggests that the level of mitochondrial activity in the *Scd-1* ^-/-^ group was higher than that in the WT group and that OA has the ability to partially inhibit the extent of mitochondrial activity. No disparity in lipid droplet content was observed in Teffs between the *Scd-1* ^-/-^ and WT groups. However, the inclusion of OA during cell culture led to an increase in the lipid droplet content in Teffs of the *Scd-1* ^-/-^ group (**Figure 5H**).

In summary, the Teff mitochondrial oxidative phosphorylation function is heightened in the *Scd-1* ^-/-^ group in comparison to the WT group. This heightened activity could be attributed to the increased expression of additional effector molecules (GZMA, GZMB, GZMC, PRF, IFN-γ). The findings of Zhai et al. (2021) were consistent with this observation. The SRC of Teffs in the *Scd-1* ^-/-^ group was notably lower than that in the WT group. When faced with stimulation, Teffs in the WT group may have demonstrated better adaptability and stress resistance. OA decreased the mitochondrial metabolic activity of Teffs in the *Scd-1* ^-/-^ group, thereby protecting the cells from excessive oxidative stress and damage.

### 3.6 OA can alleviate the inflammatory levels of endotoxemia mediated by LPS

**Figure 6:**
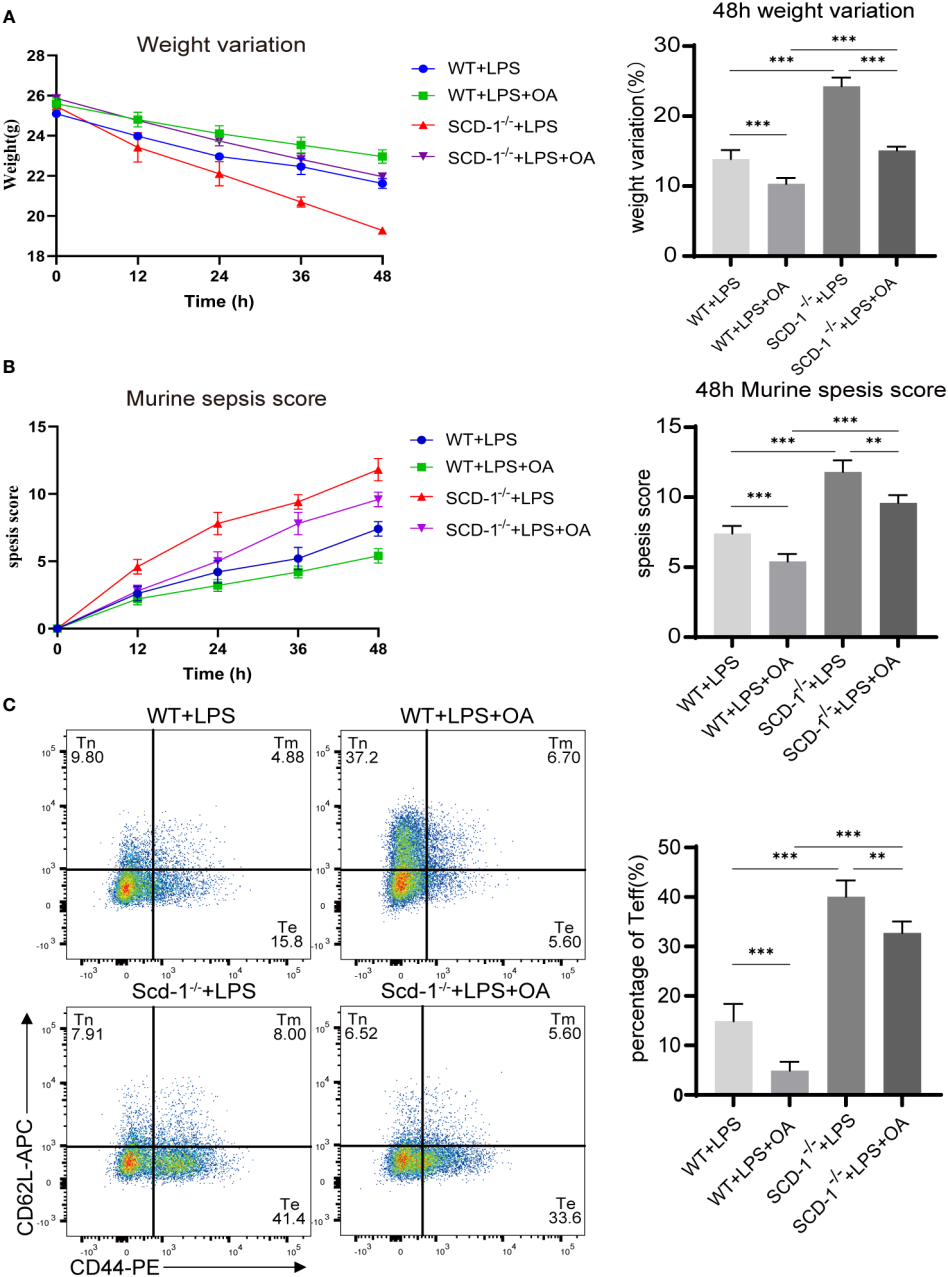
Supplement of OA partially ameliorates the severity of endotoxemia in *Scd-1* ^-/-^ mice. **(A)** Weight changes of four groups of mice 48h after LPS injection (n=5). **(B)** MSS scores of four groups of mice 48h after LPS injection (n=5). **(C)** Proportions of Teff cells in the spleen of four groups of mice 48h after LPS injection (n=5). The four groups are the WT endotoxemia group, WT endotoxemia + OA group, *Scd-1* ^-/-^ endotoxemia group, and *Scd-1* ^-/-^ endotoxemia + OA group. The results presented as mean + SEM. Each group had 5 mice. **, P<0.01; ***, P<0.001.

To examine the impact of Scd-1 and its derivative OA on inflammatory conditions, we created a model of LPS-induced endotoxemia in both WT and *Scd-1* ^-/-^ mice. The experimental group that received OA intervention was orally administered phosphate-buffered saline plus OA daily for 14 days prior to the establishment of the disease model, while the control group was administered phosphate-buffered saline. Throughout this time period, the mice were evaluated every 12 h using the murine sepsis score (MSS), and their weights were measured in accordance with the approach outlined by Shrum et al. (2014). The group of mice without Scd-1 showed the most notable decrease in weight and the highest MSS score after 48 h, suggesting that the absence of Scd-1 exacerbates endotoxemia symptoms. Our findings revealed that prior administration of OA mitigated the reduction in body weight and lowered the MSS in the intervention group, suggesting that early consumption of OA can effectively alleviate endotoxemia-related lesions. However, mice in the *Scd-1* ^-/-^ endotoxemia + OA group experienced greater weight loss than those in the WT endotoxemia + OA group and had higher MSS scores. This suggests that, while compensation for OA can partially alleviate endotoxemia symptoms, it cannot fully eliminate the worsening of endotoxemia symptoms due to the absence of *Scd-1* (**Figures 6A, B**).

**Figure 7:**
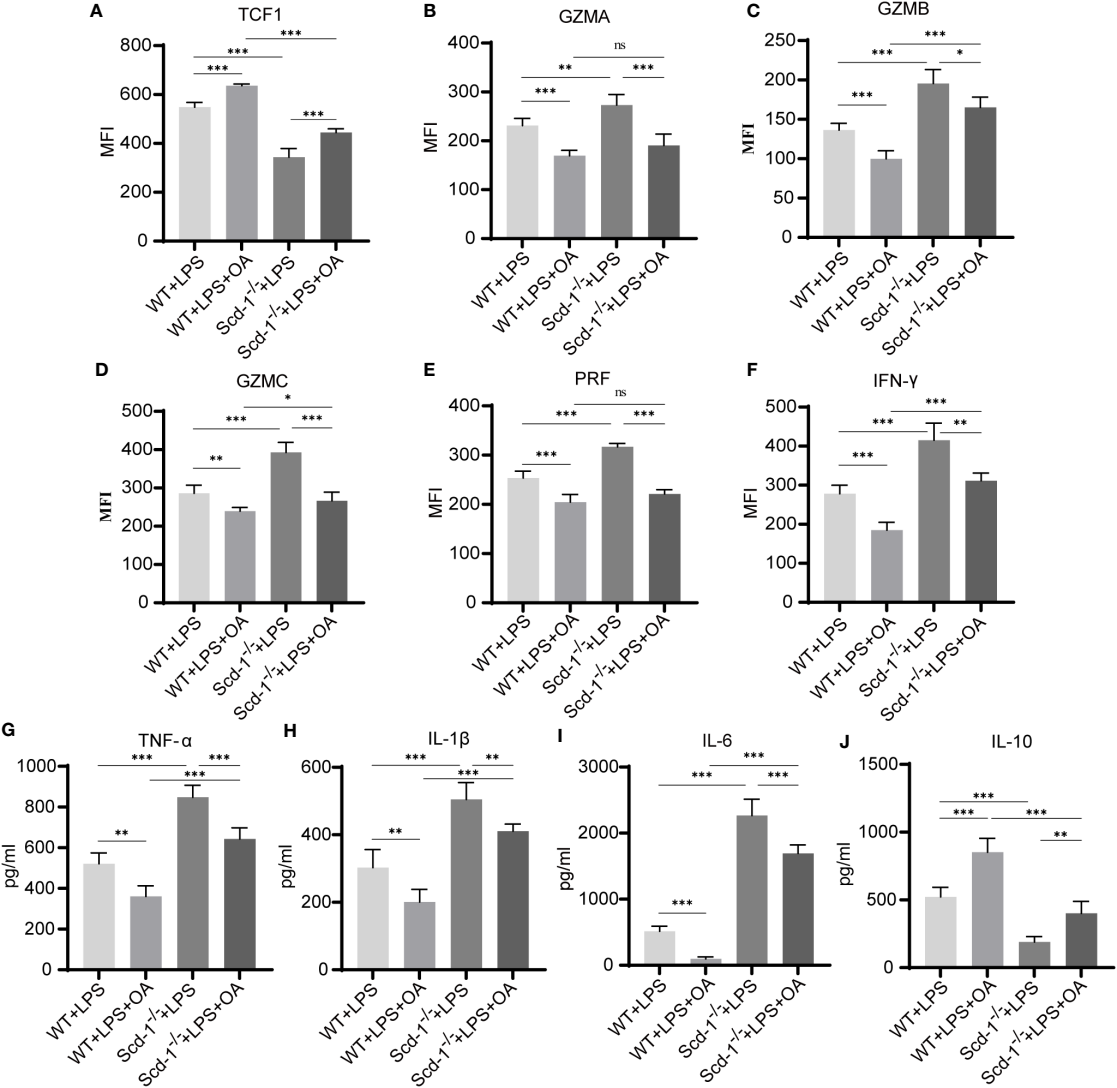
Supplement of OA partially inhibits endotoxemia inflammation in *Scd-1* ^-/-^ mice. **(A)** Expression of Tcf1 in Teff cells of four groups of mice 48h after LPS injection (n=5). **(B-F)** Expression levels of GZMA, GZMB, GZMC, PRF, and IFN-γ in Teff cells of four groups of mice 48h after LPS injection (n=5). **(G-J)** The levels of TNF-α, IL-1β, IL-6, and IL-10 in the serum of four groups of mice 48h after LPS injection (n=5). The four groups are the WT endotoxemia group, WT endotoxemia + OA group, *Scd-1* ^-/-^ endotoxemia group, and *Scd-1* ^-/-^ endotoxemia + OA group. The results presented as mean + SEM. Each group had 5 mice. *, P<0.05; **, P<0.01; ***, P<0.001. ns, not significant.

We observed that LPS-induced endotoxemia resulted in an elevated proportion of Teff cells in the spleen (**Supplementary Figure 3**). The *Scd-1* ^-/-^ endotoxemia group exhibited the highest percentage of Teffs in the spleen, whereas the WT endotoxemia + OA group had the lowest Teff proportion. The proportion of Teffs in the OA-treated groups that intervened with OA was substantially reduced. In line with prior findings, this demonstrated that the absence of Scd-1 promoted the inclination of CD8^+^ T cells to differentiate into the Teff subset, whereas the addition of OA partially hindered the differentiation of CD8^+^ T cells into the Teff subset (**Figure 6C**). In relation to the transcription factor Tcf1, it was observed that the *Scd-1* ^-/-^ endotoxemia group, which had the highest proportion of Teff, exhibited the lowest expression of Tcf1. Conversely, the WT endotoxemia + OA group, which had the lowest proportion of Teffs, exhibited the highest Tcf1 expression. Additionally, the groups that received OA supplementation displayed higher Tcf1 expression than those that did not receive OA supplementation (**Figure 7A**). In terms of the expression of effector molecules in Teff, the *Scd-1* ^-/-^ endotoxemia group had the highest expression level of effector molecules, while the WT endotoxemia + OA group had the lowest expression level. Treatment with OA resulted in the downregulation of effector molecule expression in Teff (**Figures 7B–F**). TNFα, IL-1β, and IL-6 serve as pro-inflammatory cytokines, and act as indicators of high-inflammatory phase (Faix, 2013). The levels of inflammatory mediators were highest in the blood of endotoxemic *Scd-1* ^-/-^ mice and lowest in the blood of endotoxemic WT mice administered with OA. Compared to the groups that did not use OA, the use of OA reduced the levels of pro-inflammatory cytokines in the serum (**Figures 7G–I**). However, the opposite trend was observed for the anti-inflammatory factor, IL-10 (**Figure 7J**).

These findings suggest that elimination of Scd-1 encourages the transformation of CD8^+^ T cells into the Teff subset and boosts their effector capacity, potentially contributing to the heightened inflammatory reaction observed in endotoxemic mice lacking Scd-1. OA, which is derived from Scd-1, can partially hinder the transformation of CD8^+^ T cells into the Teff subgroup, decrease the expression of active substances, and alleviate inflammation associated with endotoxemia.

### 3.7 Scd-1 deficiency increases resistance to viral infection

**Figure 8:**
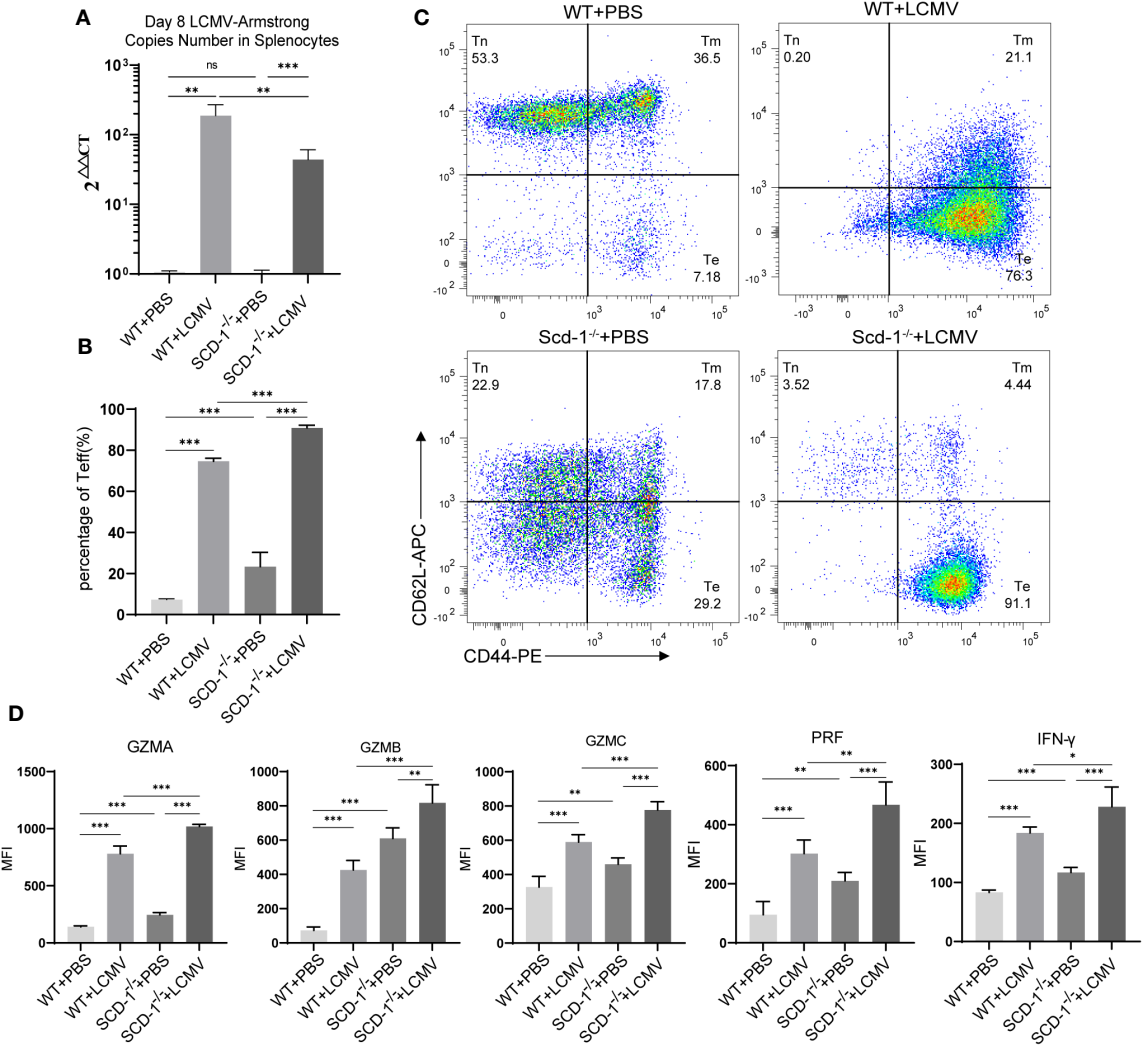
Knocking out Scd-1 increases resistance to viral infection. **(A)** LCMV-Armstrong load in splenocytes from four groups of mice at the 8th day after LCMV injection (n=5). **(B, C)** Proportions of Teff cells in the spleen of four groups of mice at the 8th day after LCMV injection (n=5). **(D)** Expression levels of GZMA, GZMB, GZMC, PRF, and IFN-γ in Teff cells of four groups of mice at the 8th day after LCMV injection (n=5). The four groups are the PBS injection WT group, LCMV injection WT group, PBS injection *Scd-1* ^-/-^ group, and LCMV injection *Scd-1* ^-/-^ group. The results presented as mean + SEM. Each group had 5 mice. **, P<0.01; ***, P<0.001. ns, not significant.

In order to exclude the potential influence of innate immune cells as much as possible and to better reflect the role of CD8^+^ T cells, here we utilized the LCMV-Armstrong strain to induce an acute systemic viral infection in mice. At the 8th day after intraperitoneal injection of 2×10^5 plaque-forming units of LCMV-Armstrong in both WT and *Scd-1* ^-/-^ mice, we found that the virus copies number in the spleen of *Scd-1* ^-/-^ mice was lower than that in the WT group (**Figure 8A**). The proportion of Teffs in the spleen of LCMV-infected *Scd-1* ^-/-^ mice is slightly higher than in the LCMV-infected WT group, which is the reason for the lower virus burden in the *Scd-1* ^-/-^ group (**Figures 8B, C**). Afterwards, we measured the expression levels of these four groups of CD8^+^ T cell effector molecules, and consistent with the previous results, the *Scd-1* ^-/-^ group showed higher expression levels of effector molecules compared to the WT group, regardless of whether they were infected with LCMV or not (**Figure 8D**).

## 4 Discussion

Although the field of immune metabolism has rapidly progressed, our understanding of the functions of fatty acids in adaptive immunity remains limited. This is mainly because of the highly diverse and multifunctional nature of fatty acids, which makes it difficult to elucidate their immune functions. Most previous studies have focused on PUFA and their derivatives (Kosaraju et al., 2017; Crouch et al., 2019). Our study provides additional insights into the relationship between Scd-1 and its product, MUFA, and adaptive immunity. The impact of Scd-1 and its product OA on CD8^+^ T-cell differentiation, functional status, and metabolic state has important implications for understanding and controlling adaptive immunity in diseases.

Our results indicate substantial differences in MUFA content among CD8^+^ Tnaïve, Teff, and Tmem. The Teff subgroup had substantially elevated levels of multiple MUFA compared with the control and Tmem groups, especially OA and PO. In line with this, the enzyme Scd-1, which is responsible for generating OA and PO, was expressed at considerably higher levels in Teffs than in Tnaïve and Tmem. This suggests that MUFA and Scd-1 have crucial impacts on the cellular status and function of Teff cells. The comprehension of fatty acids, which are constituents of cellular membranes, not only affects the flexibility of membranes but also has a considerable influence on the differentiation and functioning of T cells. In addition, they serve as a means of storing energy for cellular metabolism (Lochner et al., 2015). According to our data, the percentage of Teff in *Scd-1* ^-/-^ mice was greater than in WT mice, and they exhibited increased expression and secretion of cytotoxic effector cytokines. *In vitro*, the trend of CD8^+^ T cell differentiation toward Teffs caused by Scd-1 knockout and enhanced Teff cell effector function could be partially reversed by OA. Studies have demonstrated that omega-3 polyunsaturated fatty acids and their derivatives possess the ability to hinder the transformation of CD4^+^ T cells into Th1 and Th17 cells (Chiurchiù et al., 2016). In terms of the impact on T cell function, Omega-3 polyunsaturated fatty acids can weaken the secretion of certain cytokines by CD4^+^ and CD8^+^ T cells, including IFN-γ, IL-17, TNF-α, and IL-2 (Chiurchiù et al., 2016; Kim et al., 2018).

T cell immunity relies heavily on cellular metabolism, specifically on metabolic pathways such as glycolysis, fatty acid oxidation, and mitochondrial metabolism, as they are closely associated with the activation, differentiation, and effector functions of T cells (Bantug et al., 2018). Fatty acid oxidation is the primary source of energy for CD8^+^ naïve T cells. As they differentiate into effector T cells, their metabolic pathways shift toward glycolysis to sustain their effector functions. In contrast, memory T cells rely predominantly on fatty acid oxidation to fulfill their energy requirements (Gerriets and Rathmell, 2012; van der Windt and Pearce, 2012). When Scd-1 was knocked out, Teff cells exhibited increased mitochondrial oxidative phosphorylation and glycolysis, which may have been caused by the expression of more effector molecules. Furthermore, elimination of Scd-1 greatly diminished SRC, suggesting that Scd-1 plays a crucial role in preserving ATP generation under stressful conditions and cannot be restored by Scd-1 derivatives. In the *Scd-1* ^-/-^ group, OA reduced the metabolic activity of Teff in the mitochondria, thereby protecting cells from excessive oxidative stress and harm. Moreover, the inclusion of three additional fatty acids may also decrease the oxidative phosphorylation level of Teff in the *Scd-1* ^-/-^ group, potentially because of the identical byproducts they generate. However, this phenomenon requires further investigation.

In the LPS-induced endotoxemia model, our data indicate that knockout of Scd-1 exacerbates endotoxemia symptoms and enhances the inflammatory response, while pretreatment with OA can alleviate symptoms and reduce inflammation levels. In line with the findings of Medeiros-de-Moraes et al. (2018), OA intervention could increase the level of IL-10 and decrease levels of TNF-α and IL-1β in septic mice. One possible immunomodulatory mechanism of OA is the inhibition of CD8^+^ T cell differentiation into effector T cells and the subsequent downregulation of effector molecule expression, which could contribute to a reduction in inflammatory responses. Considering that we did not employ conditional knockout mice for Scd-1, it was not possible to completely eliminate the influence of Scd-1 deficiency on other immune cells. Previous studies have revealed that in cases of sepsis, there is a reduction in the overall number of CD8^+^ T cells, including all subgroups, such as CD8^+^ Tnaïve, Teff, and Tmem cells (Choi et al., 2017; Jensen et al., 2018). It has also been shown that 2 days after the organism is attacked by endotoxin, the percentage of Ki67^+^ CD8^+^ T cells are significantly increased both in patients with sepsis or endotoxemia(Rodriguez-Rosales et al., 2019). During our examination of endotoxemic *Scd-1* ^-/-^ mice, we noticed an increase in the ratio of Teff cells, and this may be related to the level of inflammation as well as its duration.

OA is an Omega-9 fatty acids, and notably a diet rich in OA has a positive anti-inflammatory effect on many inflammation-related diseases such as retinitis, pneumonia, hepatitis, and intestinal inflammation (Bettadahalli et al., 2020; Cariello et al., 2020; Yu et al., 2020; Leone et al., 2021). OA regulates the immune system by activating various immune cells. Under different physiological and pathological conditions, it can alter the production of substances that cause inflammation, control the entry of certain white blood cells, and adjust the creation of factors that promote the growth of blood vessels, all of which help reduce inflammation (Farag and Gad, 2022). According to earlier studies, nitrooleic acid decreases the transcriptional activity of NFAT, which in turn controls the synthesis of pro-inflammatory cytokines produced by stimulated T cells and manages immune responses mediated by T cells (Bago et al., 2023). Gaining a comprehensive understanding of how OA affects various immune cells will facilitate doctors to further optimize and implement OA supplements for the treatment of various diseases.

There is a point in our study that confuses us: in WT mice, Teff cells have higher levels of Scd-1 and OA compared to Tnaïve and Tmem cells. However, when Scd-1 is knocked out, CD8^+^ Tnaïve cells seem to preferentially differentiate into Teff. Although we have demonstrated differences between WT and Scd-1^-/-^ Teff in terms of cytokine secretion and mitochondrial metabolism, we have not investigated other aspects such as proliferation and apoptosis in depth. Further exploration is needed in future studies to delve into these areas. In subsequent studies, we will also consider using other markers in addition to CD44 and CD62L to more accurately define Teff, which may help address the issues mentioned above. Since the mice used in this study were not conditional knockout mice, even though there were no significant differences in the proportions of several immune cells observed in the spleen (**Supplementary Figure 2D**), it is still difficult to rule out other interference.

In summary, gaining a more profound and all-encompassing understanding of the metabolism of lipids in immune cells and their controlling effects on cell function, growth, and distinction will aid in the creation of novel therapeutic approaches to control the immune response, resulting in an enhanced prognosis and potentially even a remedy.

## Data availability statement

The RNA-seq data presented in the study are deposited in the GEO repository, accession number GSE253770.

## Ethics statement

The animal study was approved by Zhongshan Hospital, Fudan University Ethical Committee. The study was conducted in accordance with the local legislation and institutional requirements.

## Author contributions

YL: Investigation, Methodology, Data curation, Writing – original draft. XL: Investigation, Data curation, Writing – original draft. HS: Formal analysis, Writing – original draft. JG: Investigation, Writing – original draft. YY: Methodology, Writing – original draft. LJ: Formal analysis. LS: Investigation, Data curation. YC: Investigation, Writing – original draft. FL: Visualization, Software, Writing – original draft. XY: Funding acquisition, Project administration, Writing – review & editing.
