# Supplementary material for: Scd-1 deficiency promotes the differentiation of CD8+ T effector
Source: Front Cell Infect Microbiol. 2024 Feb 6;14:1325390. doi: 10.3389/fcimb.2024.1325390 (PMC10876803; doi:10.3389/fcimb.2024.1325390)

**Supplementary Information**

**Scd-1 Deficiency Promotes the Differentiation of CD8^+^ T Effectors**

Yiwei Lin†, Xushuo Li†, Haojie Shan, Jie Gao, Yanying Yang, Linlan Jiang, Lu Sun, Yuwen Chen, Fangming Liu, Xiaowei Yu*

**Figure S1, related to Figure1**


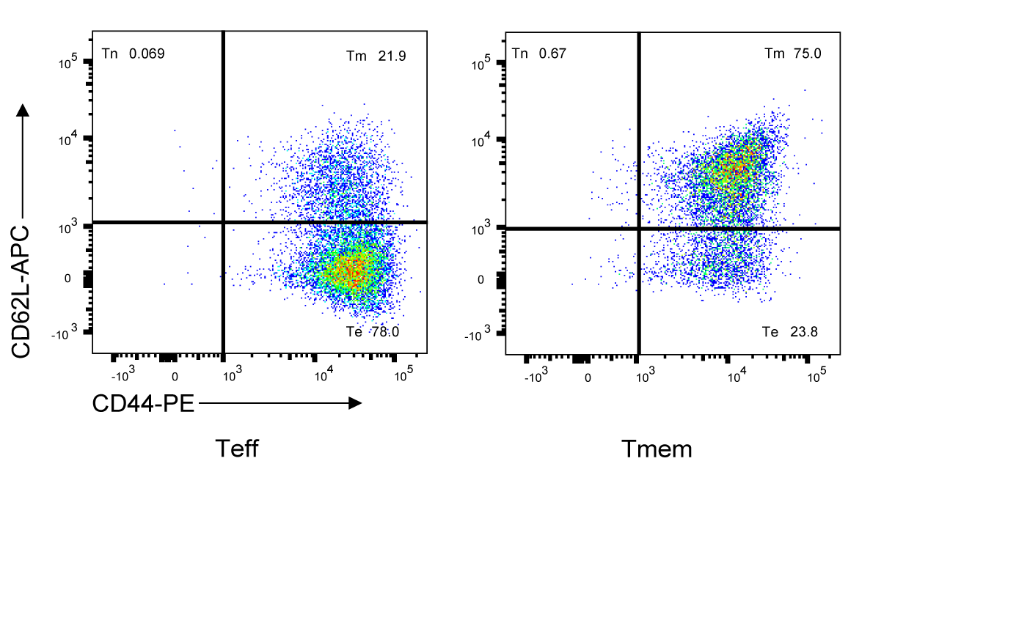


**Figure S1.** **Flow cytometry of Teff and Tmem in culture, related to Figure1**

Flow cytometry of Teff (CD44^hi^ CD62L^lo^) and Tmem (CD44^hi^ CD62^hi^) in culture, the incubation process was as described in materials and methods.

**Figure S2, related to Figure2**


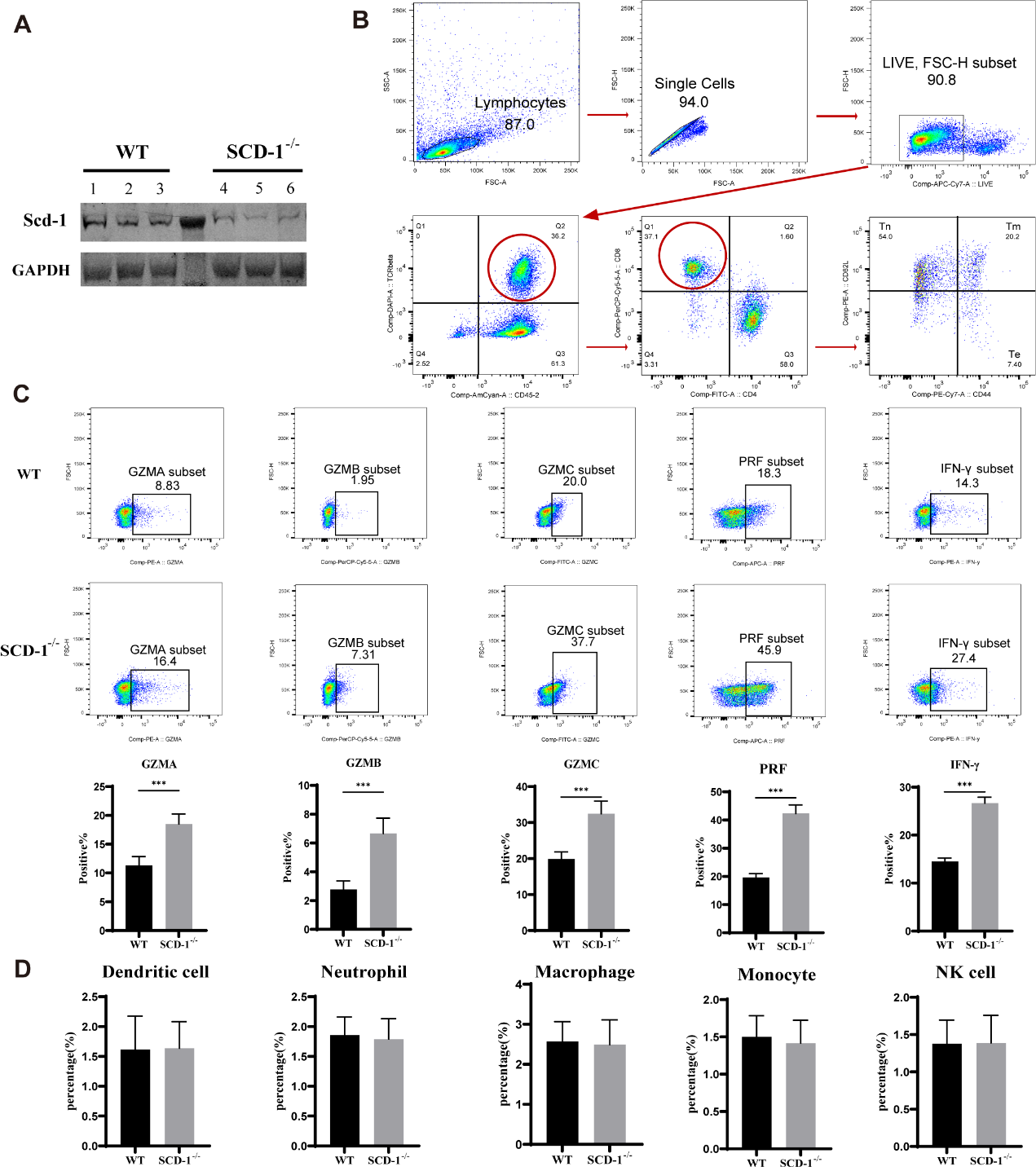


**Figure S2.** **Comparison of the proportions of several immune cells in the spleens of WT and *Scd-1*^-/-^ mice, related to Figure2**

1. Immunoblot analysis of Scd-1 in CD8^+^ T cells of WT and *Scd-1^-/-^* mice by immunoblot.
2. The gating steps for Tnaïve (CD44^lo^ CD62L^hi^), Teff (CD44^hi^ CD62L^lo^), and Tmem (CD44^hi^ CD62^hi^) in flow cytometry.
3. The real facs plots for GZMA, GZMB, GZMC, PRF, and IFN-γ in WT and *Scd-1^-/-^* mice (n=5).
4. The proportion of dendritic cell(CD45.2^+^ CD11c^+^ I-A/I-E^+^), neutrophil(CD45.2^+^ CD11c^-^ CD11b^+^ Ly6G^+^), macrophage(CD45.2^+^ CD11c^-^ CD11b^+^ Ly6G^-^ Ly6C^lo^), monocyte(CD45.2^+^ CD11c^-^ CD11b^+^ Ly6G^-^ Ly6C^hi^), NK cell(TCRβ^-^ CD45.2^+^ Nkp46^+^ CD49b^+^) in the spleen of WT and *Scd-1^-/-^* mice (n=5).

The results presented as mean + SEM. ***, P＜0.001.

**Figure S3, related to Figure6**


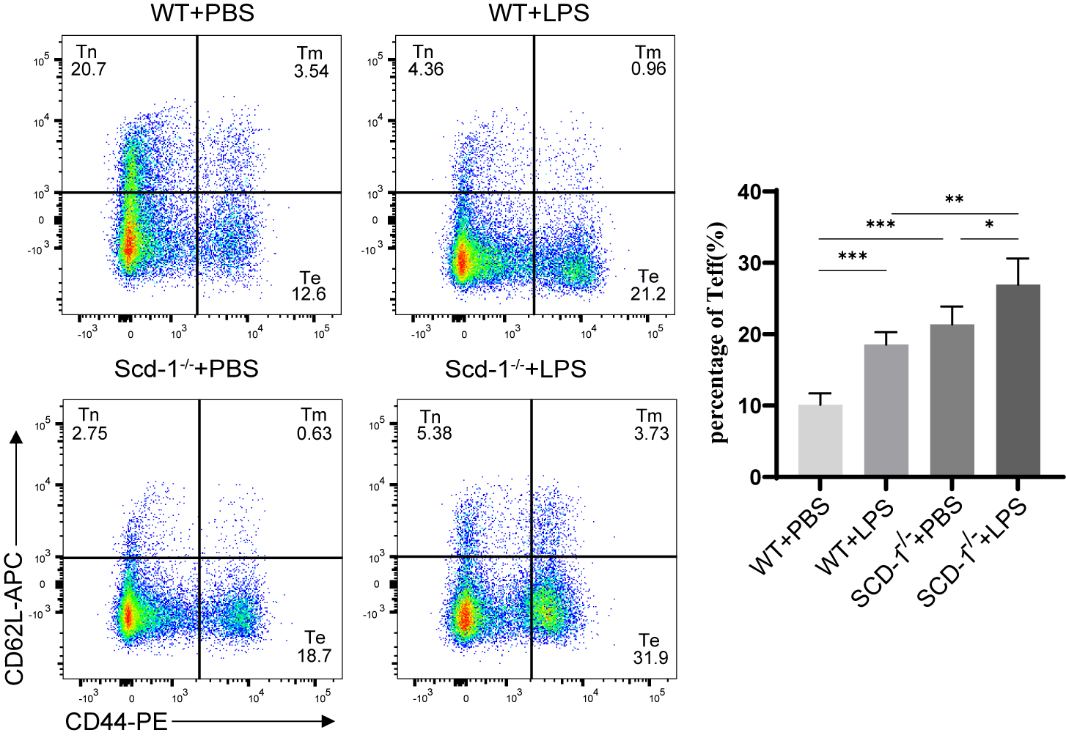


**Figure S3.** **The proportion of Teff in the spleen after 3mg/kg LPS injection in WT and *Scd-1^-/-^* mice at 48 hours, related to Figure6**

Proportions of Teff cells in the spleen of four groups of mice 48h after LPS injection (n=5).

The four groups are the WT + PBS group, WT + LPS group, *Scd-1*^-/-^ + PBS group, and *Scd-1*^-/-^ + LPS group. The results presented as mean + SEM. *, P＜0.05; **, P＜0.01; ***, P＜0.001.

**TABLE S1.** **Antibody Information**

**
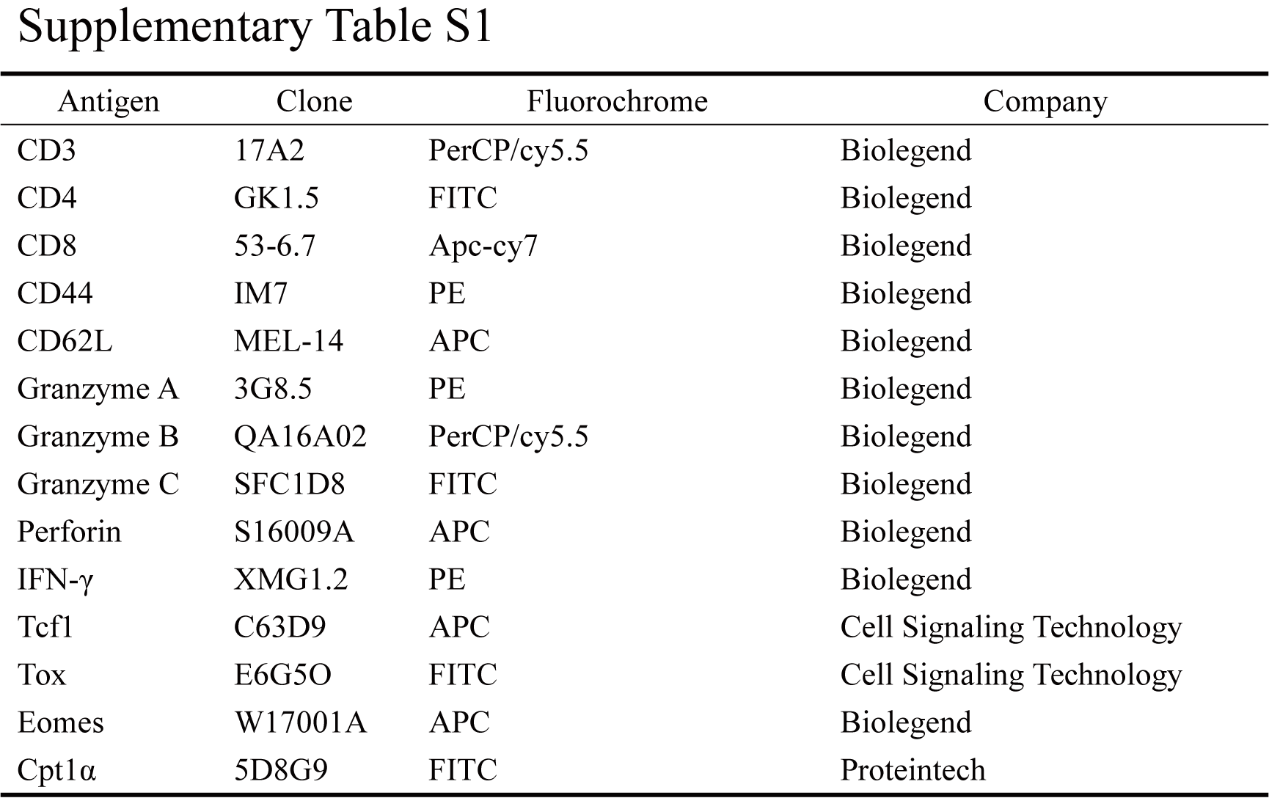
**

**TABLE S2.** **Primer Information**


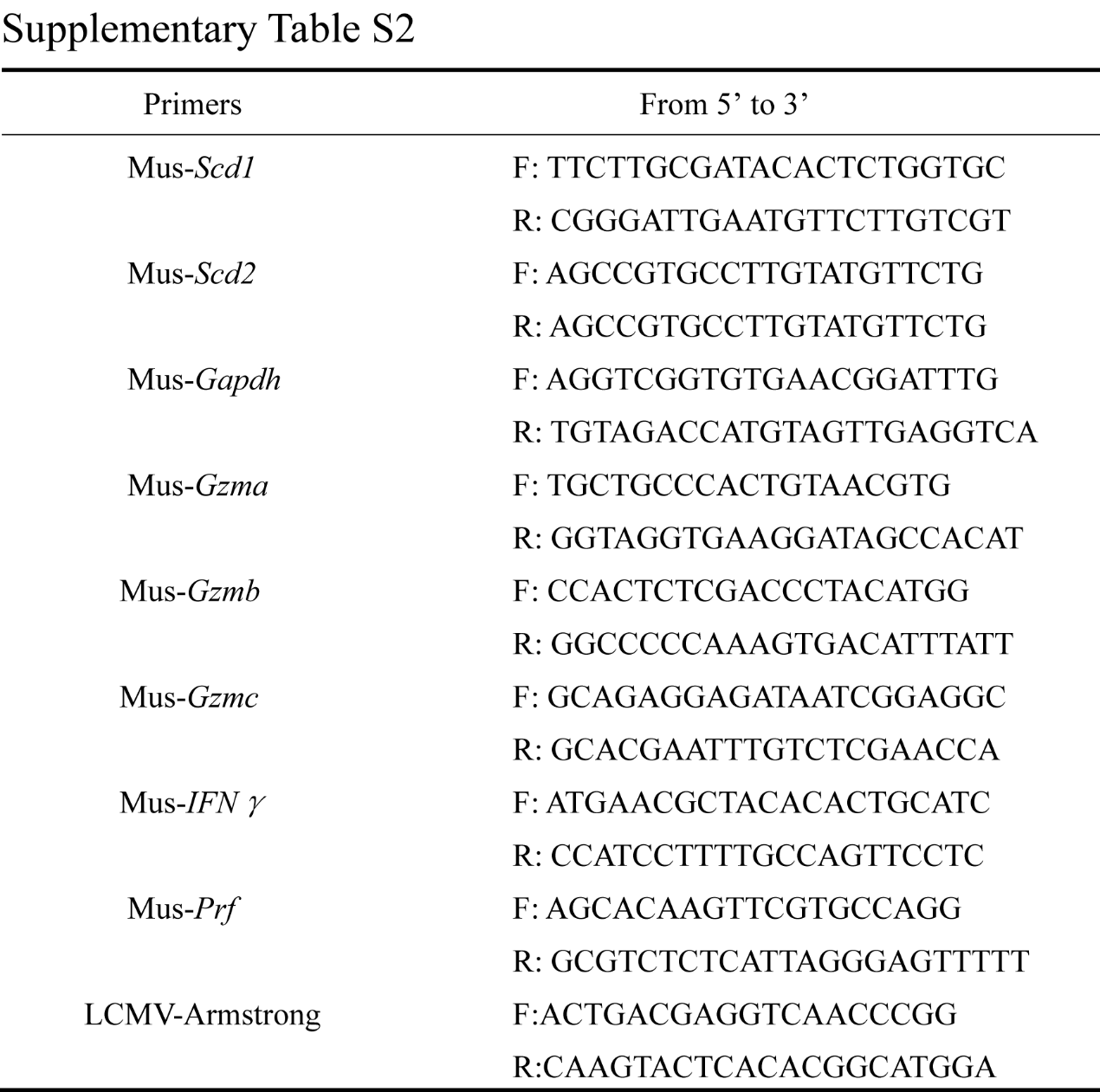

Supplement: Supplementary file 1 [file DataSheet_1.docx]
